# A population-based study of Kurdish breast cancer in northern Iraq: Hormone receptor and HER2 status. A comparison with Arabic women and United States SEER data

**DOI:** 10.1186/1472-6874-12-16

**Published:** 2012-06-22

**Authors:** Majid A Runnak, Mohammed A Hazha, Hassan A Hemin, Abdulmahdi A Wasan, Rashid M Rekawt, Hughson D Michael

**Affiliations:** 1 Department of Pathology, Shorsh General Hospital, Sulaimaniyah, Iraq; 2 Division of Oncology, Hewa Hematology and Oncology Hospital, Sulaimaniyah, Iraq; 3 Office of the Director of Health, Sulaimaniyah Governate, Sulaimaniyah, Iraq

**Keywords:** Breast cancer, Hormone receptors, HER2, Estrogen receptors, Middle East, Kurdish, Arabic, Iraqi women

## Abstract

**Background:**

Hormone receptor (HR) and HER2 expression predict the therapeutic response and prognosis of breast cancer. In the Middle-East, breast cancer is diagnosed at a young age, and Arabic women are reported to have a low frequency of HR positive tumors. This study investigates HR and HER2 expression among Kurdish and Arabic women.

**Methods:**

During 2008–2010, the Sulaimaniyah Directorate of Health records identified 514 Sulaimaniyah Kurdish women, 227 Kurdish women of other Governates, and 83 Arabic women with a first diagnosis of breast cancer. The breast cancers of 432 women had immunohistochemistry (IHC) performed for estrogen and progesterone receptors (ER and PR) and HER2. Age specific and age standardized incidence rates were calculated for Sulaimaniyah Kurds. Results were compared with Egypt and with United States (US) SEER data.

**Results:**

The median patient age was 46 years and 60.4% were < 50 years old. Tumors of 65.2% of women were ER+/HER2- with the rate increasing to 78.3% in patients ≥ 60 years old in proportions similar to US whites. The total annual age standardized incidence for breast cancer among Sulaimaniyah Kurds was 40.5/100,000 women, a rate similar to Egypt but much lower than the US. By HR/HER2 subtype, the highest age specific incidence rates were 16.4 and 45.4/100,000 for ER+/PR+/HER2- tumors in women < 50 or ≥ 50 years old, respectively (US whites: 37.7 and 226.1/100,000). Tumors of 20.4% of Sulaimaniyah women were HER2+ with annual incidence rates for ER-/PR-/HER2+ tumors of women <50 or ≥ 50 years old being 4.0 and 6.3/100,000 (US whites: 3.2 and 14.4/100,000). No significant differences in ER or HER2 status were found between Kurdish and Arabic patients.

**Conclusions:**

Compared to the US, low age standardized and age specific breast cancer incidence rates were found in Kurdish women; nevertheless, the proportional expression of HR and HER2 for both Kurds and Arabs was comparable to that of US white women. The great majority of the breast cancer was ER+/HER2- and should respond to anti-estrogen therapy.

## Background

Estrogen receptor (ER), progesterone receptor (PR), and HER2 expression of breast cancers vary by patient age, race, and ethnicity [1-6]. In the United States (US), ethnic and racial differences in hormone receptor (HR) status have been investigated in large numbers of patients through the Surveilance Epidemiology and End-Results (SEER) and Carolinas Breast Cancer Study data bases [1-8]. On average, African American breast cancer patients are younger than whites, but even at comparable ages, tumors are less frequently ER + and the clinical outcomes for African Americans are demonstrably worse than for whites [1,4,5,7,9-11].

Breast cancer can be divided into four major subtypes on the basis of HR and HER2 testing: those that are HR + (ER + and/or PR+) but HER2-, those that are HR + and HER2+, those that are HR- and HER2+, and those that are “triple negative” for ER, PR, and HER2 [7,8,10-12]. HR+/HER2- tumors are the most common subtype and account for 50% to 80% of breast cancers [1-5]. Patients with ER+/PR + tumors have a more favorable survival than women with ER-/PR- tumors and the survival advantage increases substantially with hormonal therapy [1]. HER2+ tumors comprise 5-25% and triple negatives 5-15% of breast cancer [7,8,10,13]. Patients with HER2+ and triple negative tumors tend to be younger, to have higher grade tumors at higher clinical stages, and to have shorter survivals [7,8,10,11,13].

The major importance of subtypes is that they determine the medical treatment patients receive [1,6,12]. ER and PR are strongly predictive of a response to tamoxifen, and little benefit for tamoxifen can be shown for HR- negative tumors [12]. The monoclonal antibody trastuzumab is applicable only for HER2+ tumors and improves survival when administered following anthracyclin-containing protocols that often contain a taxol [14-19]. For ER + tumors, HER2 over-expression is associated with lower ER levels and with tamoxifen resistance [12,16]. Triple negative tumors tend to be chemosensitive to taxanes, platinums, and antracyclines but are problematic because of their short disease free intervals (13).

As in most of the Middle East, breast cancer in Iraq is the most common documented cancer and is the leading cause of cancer death among women [20-24]. Studies from Saudi Arabia and Jordan have reported that Arabic women have a high proportion of ER- and PR- breast cancers that occur predominantly under 50 years of age [23,24]. It is suggested that breast cancer in the Middle-East may be unusually aggressive, and that the prognosis for individual patients is unlikely to be favorable [23,24].

While Kurds are ethnically distinct from the Arab population of Southern Iraq, our preliminary studies have found that the age specific breast cancer incidence for Kurdish women was similar to that of Egypt and Jordan [20,21]. The median age of Kurdish patients at diagnosis was 46 years, 60% of women were premenopausal, and nearly 70% of patients had stage 2 or 3 disease [20].

These findings were disquieting but indicated a basic similarity of breast cancer throughout the region. Studies on HR and HER2 in the Middle-East are limited, and the results vary widely [22-27]. Since breast cancer subtypes are indicators of the choice of and the potential response to treatment, this study was undertaken to determine the age related distribution of the breast cancer subtypes among a sizable number of Kurdish and Arabic women treated at a Northern Iraq reference center and to compare the findings with women reported in the US SEER program.

## Methods

The Sulaimaniyah Directorate of Health serves as the ethics committee for Hewa Hospital and gave permission for the research. The research was conducted according to the Helsinki Accords. In 2005, the Directorate of Health established Hewa Hematology and Oncology Hospital as the central institution for collecting data on cancer patients and coordinating cancer care in the region. In the three year period of 2008–2010, 824 women were recorded as having a first diagnosis of breast cancer. Registry information included age at diagnosis, place of residence, and record of histologic confirmation of diagnosis.

For the 824 women, 432 had breast biopsies or mastectomy specimens reviewed or analyzed at Shorsh Hospital Pathology Laboratory and immunohistochemical (IHC) assays were performed. Breast cancer patients at Hewa Hospital are evaluated and treated by 4 oncology physicians. There are no treatment guidelines for IHC testing or for treatment, and the decision to refer specimens for IHC was the choice of the attending physician. Only two of the four physicians routinely requested specimen reviews and IHC studies.

Histological sections were cut at 4 μ and stained with hematoxylin and eosin (H&E) stains and additional sections were affixed to charged slides for IHC. The breast cancers were classified by histologic type and given a Nottingham combined histologic grade. Slides for IHC were stained for ER, PR, and HER2 using antibodies, buffers and linking systems purchased from Dako™ (Dako, Denmark). ER used clone SP1, PR clone PgR 636, and HER2 polyclonal rabbit anti-human c-erbB-2. Procedures were performed with 15 minutes pressure cooker heat induced epitope retrieval in pH 9.0 TRIS target retrieval solution (TRS) for ER and pH 7.0 citrate TRS for PR and HER2. The linking/amplification system consisted of EnVision™ Systems polymer-enzyme conjugate (Dako, Denmark).

The ER and PR antibody reactions were graded semiquantitatively using the “Allred” 0 to 8 scoring system that adds scores for signal intensity (0, none; 1, weak; 2, moderate; 3, strong) and the proportion of positive tumor cell nuclei (0, none; 1 < 1%; 2, 1-10%; 3, 11-33%; 4, 34-66%; 5, 67-100%). Scores ≥ 3 were considered positive. HER2 was graded as 0 to 3+ with 3+ having strong complete membrane staining around > 30% of tumor cells. Scores of 3+ were considered over-expressed or positive and scores ≤ 2+ as negative. Fluorescence *in-situ* hybridization for HER2 amplification was not performed.

For Sulaimaniyah residents, annual age specific incidence rates per 100,000 women were calculated on the basis of the 2002 World Health Organization (WHO) census estimates for Sulaimaniyah Governate. An age standardized incidence rate was calculated for the Sulaimaniyah population using the WHO population distribution ratio. An age standardized incidence rate for Egypt was obtained from the monograph of the NIH sponsored Middle-East Cancer Consortium [21]. Incidence estimates for Sulaimaniyah and Egypt were compared with US SEER data from Atlanta, Georgia for women diagnosed with breast cancer in 2003–2004 as tabulated by Lund *et al.*[7]. The source of the population of white and African American women at risk by age for the two county Atlanta area was the 2000 US Census. For triple subtypes, age specific incidence rates for Sulaimaniyah residents < 50 and ≥ 50 years old was calculated using the one year average of each subtype multiplied by a factor of 1.91 to account for the 47.6% of patients who did not have IHC testing.

Data was entered into an Excel worksheet and analyzed with Stata™ (Stata Corp, College Station, TX) statistical software. The proportional distribution of patients among different groups was analyzed by Chi square tests. Comparisons of age among the three population groups used Dunn’s analysis of variance on ranks. Logistic regression was used to evaluate the relationships between HER2 status as the dependent variable and age, tumor grade, and ER status as independent variables. For all statistical procedures P < 0.5 was considered significant.

## Results

### General characteristics of the patient population

During 2008–2010, 824 new breast cancer patients were registered at Hewa Hospital (Table 1). IHC studies were performed on 432 (52.4%) patients. The average age at diagnosis for all patients was 47.8 ± 11.5 years with a range of 21 to 83 and a median of 46 years. Patients not having IHC were slightly older than those on whom IHC was performed, P = 0.03. For all patients, 514 were Kurdish residents of the Sulaimaniyah Governate, 227 were Kurdish from Erbil, Dohuk, or Germyan, and 83 were Arabs from outside of Sulaimaniyah. For the patients having IHC studies, 265 were Kurdish residents of Sulaimaniyah, 117 Kurdish women residing outside of the Sulaimaniyah Directorate, and 50 were Arab non-residents of Sulaimaniyah.

**Table 1:** Summary of characteristics of women with breast cancer having and not having immunohistochemistry (IHC) assays performed, Sulaimaniyah, Iraq (2008–2010)

Characteristic	IHC assays not performed		IHC assays performed		P value*
	No.	%	No.	%	
Patients (total = 824)	392	47.6	432	52.4	
Age					
20-49	216	55.1	261	60.4	0.26
50-59	100	25.5	102	23.6	
≥ 60	76	19.4	69	16.0	
Mean (range)	49.5 ± 12.0 (23–88)		47.8 ± 11.5 (21–83)		0.03
Ethnicity/residence					
Kurd/Sulaimaniyah	241	61.4	265	61.3	0.12
Kurd/not-Sulaimaniyah	91	23.2	117	27.1	
Arab/not-Sulaimaniyah	63	16.0	50	11.6	
Tumor stage					
1	36	9.2	37	8.6	0.97
2	121	30.9	136	31.5	
3	104	26.5	115	26.6	
4	28	7.1	28	6.5	
Unknown	103	26.3	116	26.9	

* Comparing the distribution of IHC tested and non-tested patients by age, ethnicity/residence, and tumor stage (*X*^2^ tests). The difference in the ages of IHC tested and non-tested subjects was compared by a K-S test for normality and equal distribution and a *t*-test.

No significant differences were seen by ethnicity and residence or tumor stage among patients with or without IHC. For patients having IHC studies, the diagnoses were invasive ductal carcinoma NOS in 401 cases (92.8%), invasive lobular carcinoma in 17 cases (3.9%), ductal carcinoma *in-situ* in 6 cases (3.9%), metaplastic carcinoma in 2 cases, secretory carcinoma in 2 cases, mucinous carcinoma in 2 cases, and tubular and invasive papillary carcinoma in one case each. All invasive lobular carcinomas were ER + and HER2-; 15 were PR + and two PR-.

### Comparisons of Kurdish and Arab women

By ethnicity and region, the average age of patients was not significantly different (Table 2). In all groups, the great majority of patients were < 50 years old. Most patients were found at stage 2 or stage 3 of their disease. This varied from 56.3% to 55.6% for the two Kurdish groups to 74% for Arabs. For all patients, only 11.7% were diagnosed with stage 1 tumors. Histologic grade was determined on 400 tumors. For 32 patients, IHC for hormone receptors and HER2 was performed on lymph node metastases or fine needle aspirations and a grade for the primary tumor was not assigned.

**Table 2:** Characteristics of women with breast cancers having immunohistochemistry studies performed and the proportional distributions of estrogen receptor (ER), progesterone receptor (PR) and HER2 according to ethnicity and residence

Characteristic	Kurdish Sulaimaniyah		Kurdish Not-Sulaimaniyah		Arab		P value*
	No.	%	No.	%	No.	%	
Age							0.56
20-49	155	58.5	76	65.0	30	60.0	
50-59	67	25.3	25	21.4	10	20.0	
≥60	43	16.2	16	13.7	10	20.0	
Mean age ± SD	48.7 ± 11.1		46.4 ± 12.3		46.1 ± 11.7		0.12
Stage							0.30
1	24	9.1	12	10.3	1	2.0	
2	86	32.5	33	28.2	17	34.0	
3	63	23.8	32	27.4	20	40.0	
4	18	6.8	7	6.0	3	6.0	
Unknown	74	27.9	33	28.2	9	18.0	
Tumor grade							0.86
I	26	9.1	12	10.3	6	12.0	
II	116	43.8	57	48.7	22	44.0	
III	100	33.7	41	35.0	20	40.0	
Not assigned	23	27.9	7	6.0	2	4.0	
ER, PR and HER2							0.98
ER +	194	73.2	89	76.1	39	78.0	
PR +	170	64.2	80	68.4	36	72.0	
HER2 +	54	20.4	29	24.8	11	22.0	
ER/PR status							0.94
ER+/PR+	163	61.5	76	65.0	35	70.0	
ER+/PR-	31	11.7	13	11.1	4	8.0	
ER-/PR+	8	3.0	4	3.4	1	2.0	
ER-/PR-	63	23.8	24	20.5	10	20.0	
ER/HER2 status							0.71
ER+/HER2-	170	64.9	74	63.8	35	70.0	
ER+/HER2+	22	8.4	15	12.9	4	8.0	
ER-/HER2-	38	14.5	13	11.2	4	8.0	
ER-/HER2+	32	12.2	14	12.1	7	14.0	
Triple negative	33	12.6	10	8.6	4	8.0	

* Comparing the distribution of patients in ethnicity/residence groups by age, tumor stage, tumor grade, and ER, PR, and HER2 status (*X*^2^ tests). The difference in ages of the ethnicity/residence groups were compared by Dunn’s analysis of variance on ranks.

The proportion of ER + tumors varied from 73.2% to 78.0% and HER2 over-expression from 20.6% to 22% with differences not being significant, P = 0.98. By population group, 61-70% of tumors were ER+/PR + and 8–11.7% were ER+/PR-. ER-/PR + tumors were found in only 2.0-3.4% of patients. The proportion of cases subclassified by ER and HER2 status and the proportion of "triple negative" tumors was similarly distributed among the ethnic/residence groups.

### Hormone receptors and HER2 by patient age, tumor stage, and tumor grade

ER/HER2 status succinctly summarizes HR and HER2 combinations (Table 3). There are eight possible ER, PR, and HER2 interactions. From the 432 patients having IHC studies, there were 48 ER+/PR- tumors (11.1%) and 13 ER-/PR + tumors (3.0%) represented in these combinations. The categories of ER+/HER2- and ER+/HER2+ tumors captures the former and omits only the 3% of tumors represented by the latter. This allows the strongest data analysis with the fewest categories of HR and HER2. For the analyses of ER/HER2 combinations with age, stage and grade, data from all ethnic/residence groups are combined. Data was initially tabulated in the ten year age ranges 20–39, 40–49, 50–59, 60–69, and 70+. No differences were seen between the ages 20–49 or in age groups over 59, and the data was consolidated into three groups 20–49, 50–59, and 60 or more years old.

**Table 3:** Estrogen receptor (ER) and HER2 status in breast cancers of all patients (Sulaimaniyah Kurds, non-Sulaimaniyah Kurds and Arabs) having immunohistochemistry studies

Characteristic	ER+/HER2-		ER+/HER2+		ER-/HER2-		ER-/HER2+		Triple negative		P value*
	No.	%	No.	%	No.	%	No.	%	No.	%	
age											0.12
20-49	154	59.9	31	12.1	36	14.0	36	14.0	30	11.7	
50-59	71	69.6	5	4.9	14	13.7	12	11.8	12	11.8	
≥ 60	54	78.3	5	7.2	5	7.2	5	7.2	5	7.2	
Stage											0.81
1	24	64.9	5	13.5	6	16.2	2	5.4	4	10.8	
2	92	68.1	10	7.4	17	12.6	16	11.9	13	9.6	
3	73	63.5	12	10.4	13	11.3	17	14.8	13	11.3	
4	16	57.1	5	17.9	5	17.9	2	7.1	4	14.3	
Grade											<0.001
I	43	97.7	0	0	1	2.3	0	0	1	2.3	
II	157	80.9	12	6.2	11	5.7	14	7.2	8	4.1	
III	65	40.6	22	13.8	38	23.8	35	21.9	33	20.6	

* Comparing the distribution of ER/HER2 status and triple negative tumors by patient age, tumor stage, and tumor grade (*X*^2^ tests).

Tumor characteristics are compared by age, tumor stage, and tumor grade.

HR and HER2 expression tended to change with age. The largest category of tumors was ER+/HER2-, and the proportion of patients with ER+/HER2- tumors increased from 59.9% at 20–49 years to 69.6% at 50–59 and 78.3% at ≥ 60 years of age. Although HER2+ tumors tended to be nearly equally distributed between ER + and ER- tumors, HER2- tumors were predominantly found in those that were ER+. The proportion of patients with HER2+ tumors was 26.1% at 20–49 years of age and 14.4% at ≥ 60 years of age. Because of the relatively few patients ≥ 60 years old, these age differences tended toward but did not achieve significance, P = 0.12.

No relationship was found between ER/HER2 status and tumor stage, but by tumor grade, ER+/HER2- tumors were predominantly found in pathologic grades I or II; while, HER2+ and ER-/HER2- as well as triple negative tumors were predominantly grade III. In logistic regression with HER2 status as the dependent variable and age at diagnosis, ER status, and tumor grade as independent variables, HER2 was significantly related to a negative ER status (P < 0.001) and higher tumor grade (P < 0.001) but not to age (P = 0.48).

### Breast cancer incidence and HR/HER2 combinations in the Kurdish population of sulaimaniyah. A comparison with Egypt and US SEER data

For the 514 patients who were Sulaimaniyah residents, the age standardized incidence of breast cancer was 40.5 per 100,000 women (Table 4). The age specific incidence rate began to peak at 40–44 years of age and remained somewhat stable until age 60 when it started to decline. At age 70–74, the rate was similar to that of 35 to 39 year olds; while, at 75 years of age and above, the rates were one-half those seen among 30–34 year old women.

**Table 4:** Age specific and age standardized breast cancer incidence rates per 100,000 Kurdish women residents of the Sulaimaniyah Governate

Age	Frequency	Annual average	Female population	Age Incidence	WHO adjustment	Adjusted Incidence
0-4	0	0	112,952	0	8.86	0
5-9	0	0	101,995	0	8.69	0
10-14	0	0	115,164	0	8.60	0
15-19	0	0	105,537	0	8.47	0
20-24	2	0.7	82,206	0.9	8.22	0.1
25-29	10	3.3	80,746	4.1	7.93	0.3
30-34	29	9.7	62,706	15.5	7.61	1.2
35-39	66	22.0	37,634	58.5	7.15	4.2
40-44	99	33.0	26,768	123.3	6.59	8.1
45-49	80	26.7	23,502	113.6	6.04	6.9
50-54	66	22.0	20,256	109.1	5.37	5.9
55-59	60	20.0	15,200	131.6	4.55	6.0
60-64	35	11.7	14,197	82.4	3.72	3.1
65-69	31	10.3	12,123	85.0	2.96	2.5
70-74	21	7.0	12,419	56.4	2.21	1.3
75-79	6	2.0	5,607	35.7	1.52	0.5
80+	9	3.0	10,363	28.9	1.55	0.4
Total	514				100.00	40.5

Age adjusted incidence rates are calculated using the World Health Organization (WHO) adjustment ratio.

The average age of Sulaimaniyah residents was similar to Egyptians but was 12 years younger than US whites and 8 years younger than African Americans. Nearly 60% of Sulaimaniyah residents were under 50 years old compared to just 21.7% and 31.7% for the two US groups. Despite the younger age, 73.2% of Sulaimaniyah women had ER + tumors compared to 81.1% of US whites. ER + rates for Egyptians and African Americans were similar at 65.0 and 65.5%, respectively. Tumors were HER2+ for 20.4% of Sulaimaniyah women and 25.1% of Egyptians, higher rates than the 13.2% and 16.0% for US white and African American women, respectively.

The triple subtypes as provided in Table 5 were chosen for a comparison of HR and HER2 combinations, because results for these subtypes were recently published by Lund *et al.*[7] from a US SEER based analysis of American women in Atlanta, Georgia. The distribution of the subtypes was similar in Kurdish, US white, and African Americans except for two categories. The most common ER+/PR+/HER2- subtype occurred in a much higher proportion of US whites than either Kurds or African Americans. In contrast, the triple negative ER-/PR-/HER2-subtype was found in 26.8% of African Americans, twice the rate of 12.4% observed for both Kurds and US whites.

**Table 5:** Comparisons of Sulaimaniyah Kurdish breast cancer patients with Egyptian and with US white and African American women by age, hormone receptor (HR) and HER2 status, and age standardized and age specific incidence rates

	Sulaimaniyah		Egypt		US white		African Amer.	
Characteristics	No.	%	No.	%	No.	%	No.	%
Patients	265		203^a^		967		814	
Age Mean	48.7 ± 11.1		51.3 ± 10.8		61.2 ± 14.6		56.9 ± 13.4	
<50	155	58.5	92/172	53.5	210	21.7	258	31.7
≥50	110	41.5	80/172	46.5	767	78.3	556	68.3
HR and HER2								
ER+	194	73.2	132	65.0	732/903	81.1	506/773	65.5
PR+	170	64.2	89	43.8	636/901	70.6	426/771	55.3
HER2+	54	20.4	51	25.1	108/821	13.2	110/689	16.0
Triple subtypes								
ER+/PR+/HER2-	145	54.7	-	-	605/814	74.3	394/687	57.4
ER+/PR+/HER2+	15	5.7	-	-	67/”	8.2	60/”	8.7
ER-/PR-/HER2+	29	10.9	-	-	41/”	5.0	49/”	7.1
ER-/PR-/HER-	33	12.4	23	11.3	101/”	12.4	184/”	26.8
Incidence ^b,c^								
Total	40.5		53.9 ^d^		116.0		82.7	
ER+/PR+/HER2-								
<50	16.4		-		37.7		28.1	
≥50	45.5		-		226.1		179.2	
ER+/PR+/HER2+								
<50	2.6		-		7.6		7.4	
≥50	1.3		-		20.5		19.5	
ER-/PR-/HER2+								
<50	4.0		-		3.2		5.7	
≥50	6.3		-		14.4		16.9	
ER-/PR-/HER-								
<50	3.8		-		8.7		15.7	
≥50	10.0		-		36.4		69.0	

^a^ The Egyptian patients include 5 males.

^b^ Total incidence consists of age standardized incidence rates per 100,000 women using the WHO adjustment ratio.

^c^ For triple subtypes, annual incidence rates per 100,000 women are age adjusted for their respective populations. For Sulaimaniyah, the breast cancers identified by specific subtypes are multiplied by a factor of 1.91 on the assumption that untested patients will have the same HR/HER2 expression ratios as tested patients.

^d^ The age standardized incidence of breast cancer among Egyptian women was obtained from 1999–2001 data presented at the MECC [21].

The data for US whites and African Americans was from Atlanta, Georgia for the years 2003–2004 obtained through US SEER [7].

Compared to Sulaimaniyah residents, the age standardized incidence of breast cancer was slightly higher in Egyptians but was three times higher in African Americans and nearly four times higher in US whites. For the major ER+/PR+/HER2- subtype, the incidence for Kurdish patients < 50 years old was relatively low, although still higher than any other subtype. For patients ≥ 50 years old, the incidence of ER+/PR+/HER2- tumors increased 3-fold in Kurds and 6-fold in US whites and African Americans. ER+/PR+/HER2+ and ER-/PR-/HER2+ tumors had the lowest incidence rates of the triple subtypes in all three populations with the lowest rates being found in Kurds. The HER2 + rates changed little or increased only moderately in older patients. Triple negative tumors had the highest incidence rates after ER+/PR+/HER2- tumors in all three groups with 2.6 to 4-times higher incidences in patients ≥ 50 compared to those < 50 years old. The incidence of triple negative tumors was highest for African Americans. For Kurds, it was less than half that of US whites.

## Discussion

The study shows that Kurdish women of Sulaimaniyah have a relatively low incidence of breast cancer, but that the cancers are diagnosed at advanced stages and at an average age that is 8 to 12 years younger than women in the US. It is further shown that, at equivalent ages, the breast cancers of both Kurdish and Arabic women have HR profiles similar to that of US white women.

The young average age of breast cancer patients in the Middle-East is not the result of an increase in breast cancer in young people but because of the relatively low incidence in older women [20-22]. Egypt is thought to have the highest rate of breast cancer in the Middle-East with an age standardized incidence of 53.9/100,000 [21]. For Kurdish women, the age standardized incidence is 40.5/100,000 with both Egyptian and Kurdish estimates being less than half the rate of 116.0/100,000 seen in the US white women [20,21]. For Egyptian and Kurdish women, the age specific incidences for patients < 50 are similar or somewhat less than in the US and peak at 50–59 years of age [20,21]. These patterns contrasts markedly with the West where breast cancer steadily increases in postmenopausal women and at age 70 doubles the rates seen at 50–54 years old [9,21].

The most comprehensive studies on breast cancer HR and HER2 status in the Middle-East come from Egypt, Saudi Arabia and Jordan [22-27]. The reports emphasize the young age at diagnosis with most patients being between 40–50 years old. Outside of Egypt, the proportion of ER + cases ranges from a low of 19.9% in Saudi Arabia to 52.8% in Jordan [23,24].

An analysis of ER testing at the Gharbiah, Egypt Cancer Registry for the years 2001–2006 [22] identified 3673 breast cancer patients having a median age of 50.1 with urban women at 51.8 years being slightly older than those from rural areas at 49.2 years. It was notable that a majority of the patients (62.9%) had an unknown ER status. For rural women, the ratio of ER + to ER- tumors showed only a slight excess of ER + cases, but the urban ER + ratio was 68.9% and very similar to US white women and our Kurdish and Arabic patients. These findings have been supported by a more recent Egyptian study by Salhia *et al.*[28] and suggest that for urban Egyptian women, and by inference patients treated in Sulaimaniyah, the predominant mode of tumor development is related to estrogenic risk factors and that most tumors will be tamoxifen responsive.

The relatively high ER + rates do not mean that there is a correspondingly reduced burden of morbidity and mortality for Kurdish and Arabic breast cancer patients. In Western studies, the tumors of young patients tend to be of higher histologic grade and to have higher growth fractions than the tumors of older patients with these factors being independent of HR expression [8,29,30]. Among Sulaimaniyah patients, 57.8% of ER + tumors were found in patients < 50 years old and 12.8% were ER+/HER2+. The former, on the basis of patient age, are potentially aggressive tumors and the latter, on the basis of ER and HER2 co-expression, may be tamoxifen resistant [12,16].

The proportion of HER2+ tumors among Kurds and Arabs at 20.4% to 24.8% reflects the prevalence of high histologic grade cancers. The 21.9% of grade III tumors that were ER-/HER2+ and the 13.8% that were ER+/HER2+ are rates comparable to those seen in high grade tumors in the US and Europe [7,8,11,30]. Although, the proportions of HER2+ tumors among Kurds were higher than for US whites, the incidence of HER2+ tumors, was lower than for either US whites or African Americans.

It is the marked increase in ER+/PR+/HER2- tumors among older women that characterizes US and European populations [7]. For Sulaimaniyah Kurds, ER+/PR+/HER2- tumors are the most common triple subtype and have an incidence of 16.4/100,000 under 50 that increases to 45.5/100,000 among patients ≥ 50 years old. This contrasts with the much higher rate of 226.1/100,000 among post-menopausal US whites [7].

A Finnish study showed that over a 22 year period when the incidence of ER + tumors doubled from 44.1 to 82.3/100,000, the proportion of HER2+ tumors declined from 21.6% to 13.6% [31]. During this time, the incidence of HER2+ tumors remained stable at 12 to 13/100,000. The higher proportion but relatively low incidence of HER2+ tumors in Kurdish women appears to correspond to the Finnish data and does not indicate an increased risk of developing HER2+ breast cancers in younger women but rather a low risk of developing favorable ER+/HER2- tumors after the menopause.

Triple negative tumors that do not fall into a defined hormonal or HER2 treatment category were found in 11% of Sulaimaniyah residents and were the second most common triple subtype. This proportional rate is not excessive, and the estimated incidences of triple negative tumors for both younger and older Kurdish women were somewhat lower than US whites and considerably lower than the very high incidence among African Americans [7].

The general similarity of tumor characteristics to those of US white women suggests that breast cancer in the Middle-East resembles the Western disease and that the principles of treatment in Western medical practice may produce results comparable to the US. Improved results may be difficult to achieve in the near term. A very large proportion of Gharbiah and Sulaimaniyah patients had untested tumors and were not being provided the benefit of therapy relevant to HR or HER2 status [22,32].

In Sulaimaniyah, the failure to test was the choice of the physicians managing the patients. The Sulaimaniyah Governate supports pharmaceutical services that provide nearly all currently recommended generic drugs for breast cancer management including trastuzumab. Nevertheless, many breast cancer patients continue to be treated with surgery alone until there are recurrences or metastases. The Breast Health Global Initiative defines testing for ER status as a “basic level” of pathology evaluation for breast cancer in low and middle income countries [32,33]. The lack of studies for HR and HER2 in nearly 50% of Sulaimaniyah patients points to deficiencies in regional practice standards that appear to be common in many lower income countries but that should be remediable with suitably directed educational programs.

## Conclusions

The average age of breast cancer patients in Sulaimaniyah was 8 to 12 years younger than their US counterparts, and most patients were diagnosed at an advanced stage of their disease. Nevertheless, the frequency of the expression of ER, PR, and HER2 in breast cancers of both Kurdish and Arabic women was similar to US white women of similar age and with similar grade tumors. The findings suggest that the administration of anti-estrogenic and chemotherapy regimens appropriate for tumor HR and HER2 status could produce survivals comparable to those seen for US white women.

## Abbreviations

HR: Hormone receptors; ER: Estrogen receptor; PR: Progesterone receptor; SEER: Surveilance Epidemiology and End-Results.

## Competing interest

The authors declare that they have no interests that compete with any of the contents of the manuscript.

## Author's contributions

RAM, HAM, and HAH contributed to data collection and analysis and wrote the first draft of the manuscript. RAM was the principal contributor toward study design. RMR and WAK contributed to data collection and data analysis and revision of the manuscript. MDH contributed to study design, data analysis, and revision of the final manuscript that was reviewed and approved by all authors.

## Limitations

The study is observational and descriptive, and results are biased by an absence of standards for requesting IHC assays.

## Pre-publication history

The pre-publication history for this paper can be accessed here:

http://www.biomedcentral.com/1472-6874/12/16/prepub
